# Hot Springs of Arkansas and Their Therapeutical Indications

**Published:** 1882-06

**Authors:** J. T. Jelks

**Affiliations:** Hot Springs, Arkansas; Formerly Member of the State Medical Association of Georgia and of the Social Science Association of St. Lou’s ; Ex-President of the Board of Health of Hot Springs, and Member of the Garland Co., Ark., Medical and Surgical Society


ATLANTA

### Medical Register.

Vol. I.] JUNE, 1882. [No. 9

### Original.

### HOT SPRINGS OF ARKANSAS AND THEIR THERAPEUTICAL INDICATIONS.

By J. T. JELKS, M.D., Hot Springs, Arkansas.
Formerly Member of the State Medical Association of Georgia and of the Social Science Association of St. Lou's; Ex-President of the Board of Health of Hot Springs, and Member of the Garland Co., Ark., Medical and Surgical Society.

The Hot Springs of Arkansas are situated in a gorge of the Ozark Mountains, in Garland County, 53 miles from Little Rock and 413 miles from St. Louis. They are reached by visitors from the North over the Iron Mountain & Southern railroad as far as Malvern, and by the Hot Springs Narrow Gauge from Malvern to the springs. There are two trains daily, each way. The cars are very commodious and always nice and clean, with polite conductors and attendants. The Hot Springs consist of sixty-five distinct springs, ranging in temperature from 90° to 157° F. Some of the springs flow from the mountain side 80 to 100 feet above the valley, while others rise in the bed of the creek. With one exception they are all on the west side of Hot Springs Creek, a small stream flowing through the valley from north to south.

The town is 735 feet above the level of the sea, while the Hot Springs Mountain rises to a height of 1150 feet above the sea.

The climate is delightful. Rarely is the weather too cold for out-door exercise during the winter. In summer the days are pleasant, and as the sun goes behind West Mountain the valley gets cool; and at night a blanket is needed. Last summer (1881), as dry and hot as it was everywhere, at Hot Springs cover was necessary to comfort every night. The average temperature for the year at several resorts is given below, and Hot Springs, it will be observed, bears a favorable comparison with any of them:

St. Augustine, Fla., 69.3°; Jacksonville, Fla., 69.4°; Key West, 76.5°; Cedar Keys, 70.2°; Pensacola, 68 7°; Bermuda, for six months, November to April, 54 5°; Malaga, six months, November to April, 54.5° to 62.5°; San Diego, Cal., six months, November to April, 56.3°; Santa Barbara, Cal., six months, November to April, 55.6°; Aikin, S. C., six months, November to April, 50.1°; Santa Fe, New Mexico, 49.2°; Denver, Col., 49.2°; Colorado Springs, 46.8°; Wyoming Territory, 43.6°; St. Paul, Minn., 48 5°; Hot Springs, Ark., 65.39°. At Hot Springs the thermometer rarely goes above 92° in the shade, while at St. Louis and St. Paul it reaches as high as 106°.

Pamfilo de Narvaez and Ponce de Leon heard of these wonderful "fountains of youth," and tried to find them; DeSoto marched for these springs; and Indian traditions tell of an army of white men with tattered banners and raiment encamping on the hills not far away.

These springs had a great reputation among the aborigines for their curative properties, and we now see them occasionally bathing in the springs of their fathers. They were also a place of resort for the early settlers west of the Mississippi. Thomas Nuttall, in his Journal of Travels in the Arkansas Territory in 1819, describes the springs and Hot Springs Mountain, together with the rude accommodations of the times which the springs afforded.

The town now has a population of five thousand souls, and is rapidly improving in every respect. Titles to the property have at last passed from the government into the hands of private parties, and many handsome brick buildings are already completed or in process of construction. Accommodations for visitors are ample and embrace a wide range of prices.

The Arlington and Avenue hotels are the leading houses in the city. The price of board at these places varies from $17.50 to $21.00 per week. But good board can be obtained in nice families at $7.00 to $12.00 per week.

Where does the water come from, and what makes it hot? are questions we are often asked. I do not know. But there are many theories as to the source of the heat supply. I believe the water takes its temperature from the natural heat of the interior of the earth—possibly by the escape of steam through some crevasse into a cavern filled with water, and becoming thus heated it is thence discharged upon the earth's surface. Some claim the water is heated by electricity; others again, that the heat is produced by the contact of the water with beds of sulphate of lime.

Prof. David Dale Owen gave the following as his analysis of the waters. Taking a large but definite quantity from the different springs and evaporating to dryness, the residue gave from six to eight grammes of solid ingredients to the gallon.

| | Grammes. |
|---|---|
| Organic matter, combined with some moisture ...... | 1.16 |
| Silica, with some Sulph. Lime not dissolved by water .................................... | 1.40 |
| Bicarbonate Lime.......................................... | 2.40 |
| Bicarbonate Magnesia................................... | 0.50 |
| Chloride Potassium....................................... | 0.04 |
| Chloride Sodium .......................................... | 0 218 |
| Oxide Iron and a little Alumina.......................... | 0.133 |
| Sulph. Lime dissolved by water.......................... | 0.320 |
| Loss—Iodine (?), Bromine (?)........................... | 0 053 |
| Total.......................................................... | 6.224 |

This is the analysis of the waters after flowing from the springs, and, as stated, after being evaporated to dryness. Of course, the analysis represents nothing of the volatile ingredients. That there are volatile ingredients is demonstrated by a deposit from the vapor in the vapor rooms, from 1-20 to $\frac{3}{4}$ of an inch in thickness. I have now in my office one of the grates from a bath-house, on which this deposit reaches $\frac{3}{4}$ of an inch in thickness. It has never been analyzed, and I can not say what it contains. These grates never come in contact with the water from the springs. They are surrounded and saturated by the vapor only. This demonstrates the presence of volatile ingredients. We know the analysis of Prof. David Dale Owen gives only a negative result as far as active medicinal agents are concerned. Then we naturally turn to the volatile constituents for some of the remarkable cures effected here

What are these volatile ingredients? Dr. J. L. Gebhart, by the spectrum analysis, has obtained lines of silicon and hydrogen in combination, somewhat after the manner of the carbo-hydrogens This is a combination never before known in chemistry, I believe, and it acts, I should judge, as a stimulant. This may account for the stimulating effects of the water. Dr. J. L. Gebhart says: "It is in the volatile constituents that the chief medical virtues reside

Here, for the first time in human research, is found silicium in combination with hydrogen, distilled in nature's own laboratory, forming a medical compound that is at once stimulant, alterative and eliminant, such as no art or science has yet been able to duplicate."

Here, then, we find an ingredient which all chemical analysis necessarily fails to find. It has also been demonstrated that there is a great deal of electricity in the water. This is evidenced by the peculiar feeling experienced while bathing, as well as by the galvanometer.

May it not be possible that much of the good we derive from these waters in the treatment of nurasthenic and broken-down patients generally, is by the correlation of heat to electricity, and the latter to vital force?

For what are the waters indicated? And just here let me say I do not believe they are a specific for *anything*; but I have been able to treat successfully here many diseases that resisted ordinary remedies at home. Again, we know that here we have only chronic diseases to treat, and then, too, after they have "gone the rounds" at their homes before coming here. About seventy-five per cent. of them go away well.

Syphilis is treated successfully in all its stages—except the primary. That does not yield here any more readily than elsewhere. But the secondary and tertiary syphilis rapidly get well; not, however, by using the water alone. We are enabled to give much larger quantities of mercury and the salts of iodine here than elsewhere, and the same being rapidly washed through the system by the baths and drinking the water, do not hurt or disturb the patient as the same quantity would at the patient's home.

How do we treat syphilis? By mercury and by iodine in some one of its combinations. Some use the mercury hypodermically, others by the mouth, but I prefer to use it by inunction. It never, or very rarely, disagrees with the stomach and bowels. Many patients can use it that way who could not take it internally in any quantity. I have no experience with the hypodermic use of mercury, and consequently can not say anything concerning it from personal observation.

I usually order one drachm of the officinal mercurial ointment every night—rubbed into the *thickest* skin on the body, say the outside of the leg, thigh and hips, or on the back. This I think important, because if rubbed on the thin, tender portions of skin, as the abdomen, it very rapidly produces its effect on the bowels, and the exhibition has to be delayed.

Rheumatism—subacute and chronic—improves rapidly by the baths, aided by proper medication. Many cases I have seen have recovered without medicine. I usually give rheumatic patients quinine and benzoate of lithia. I do not know why the benzoate has a better effect than some other salts of lithia, but my experience is that it certainly does, and I use it more often than any other routine medicine for this class of cases.

Rheumatic gout, or rheumatic arthritis, does not get well here—nor, indeed, anywhere else, so far as I know. Some few cases of recovery are recorded by Niemeyer and others, but in a large practice for years I have not seen a single case recover.

Gonorrhœal rheumatism improves rapidly, and since Marcy has demonstrated the possibility of aspirating the synovial sack and thereby relieving the diseased joints of an accumulation of fluid, I expect these cases to improve more rapidly than ever.

Many cases of neurasthenia are treated here—most of them successfully. Diseases of the skin, in numberless forms, improve rapidly by judicious bathing and medication; many are cured. Diseases of the uterus and uterine appendages get well more promptly here by bathing and proper treatment than elsewhere. I have been more successful here than I could be anywhere else in the treatment of this class of diseases, possibly because I have the patients more under my control and freer from disturbing influences. Enlargements of the glands of a scrofulous nature subside with proper care and judicious bathing.

Many persons tell me they can not drink alcohol while using the hot water, and that they are better able to abandon its use while here than while at home.

Now, let me say there are many physicians here who *pay* for all the patients they get. They have no professional standing in the community, and employ the hotel drummers to bring them business, giving fifty per cent. of what they get to the "roper" or "steerer." These hotel drummers are the sharpest men in the town, and are excellent judges of human nature. When they get a visitor to their boarding houses, they find out what doctor the man desires to consult, and then tell him that the doctor is either dead, moved away, a drunkard, or will make any other false representation that may suffice to hoodwink the victim for a few hours, or until they have an opportunity to "show him around the town" and sell him to some drumming doctor. I would suggest to all who read this article that they give to their friends who may visit this place a note of introduction to some respectable resident physician here. We certainly have at least a dozen good reputable men in the town who would be an honor to the medical profession anywhere.

